# An Inflammatory Polymorphisms Risk Scoring System for the Differentiation of Ischemic Stroke Subtypes

**DOI:** 10.1155/2015/569714

**Published:** 2015-08-19

**Authors:** Elena Muiño, Jurek Krupinski, Caty Carrera, Cristina Gallego-Fabrega, Joan Montaner, Israel Fernández-Cadenas

**Affiliations:** ^1^Stroke Pharmacogenomics and Genetics, Fundació Docència i Recerca MútuaTerrassa, Mutua de Terrassa Hospital, Sant Antoni 19, Terrassa, 08221 Barcelona, Spain; ^2^Neurology Department, Mutua de Terrassa Hospital, Terrassa, 08221 Barcelona, Spain; ^3^Neurovascular Research Laboratory, Vall d'Hebron Institute of Research, Universitat Autonoma de Barcelona, Barcelona, Spain

## Abstract

Inflammation has been associated with atherothrombotic stroke and recently with cardioembolic stroke. Different genetic risk factors have been specifically associated with the subtypes of ischemic stroke (cardioembolic, atherothrombotic, and lacunar). However, there are no studies that have generated genetic risk scores for the different subtypes of ischemic stroke using polymorphisms associated with inflammation. *Methods.* We have analyzed 68 polymorphisms of 30 inflammatory mediator genes in 2,685 subjects: 1,987 stroke cases and 698 controls. We generated a genetic scoring system with the most significant polymorphisms weighted by the odds ratio of every polymorphism and taken into consideration the stroke subtype. *Results.* Three polymorphisms, rs1205 (*CRP* gene), rs1800779, and rs2257073 (*NOS3* gene), were associated with cardioembolic stroke (*p* value <0.05). The score generated was only associated with the cardioembolic stroke subtype (*p* value: 0.001) and was replicated in an independent cohort (*p* value: 0.017). The subjects with the highest score presented a cardioembolic stroke in 92.2% of the cases (*p* value: 0.002). *Conclusion.* The genetics of inflammatory markers is more closely associated with cardioembolic strokes than with atherothrombotic or lacunar strokes. The genetic risk scoring system could be useful in the prediction and differentiation of ischemic stroke; however, it might be specific to particular ischemic stroke subtypes.

## 1. Introduction

Ischemic stroke is a leading cause of death and disability worldwide [1]. Although twin and familial aggregation studies have revealed a significant genetic component involved in the etiology of stroke, not all of the genetic risk factors responsible for this heritability have been determined [2]. Classical linkage analysis approaches have reported an association of the* PDE4D* and* ALOX5AP* genes with ischemic stroke, but replication of these findings has been inconsistent [3]. In addition, candidate gene association studies have permitted the identification of several other candidate genes that may also be associated with ischemic stroke, such as* APOE*,* IL6*,* MTHFR*, and* TNFα* [2]. However, these studies have proven difficult to replicate for a number of reasons: it is because they may be false positive associations; it is because of the fact that each gene may only contribute partially to overall heritability; or it is because the studies did not study the ischemic stroke subtypes separately [4, 5].

Genome-wide association study (GWAS) approaches have highlighted the relationship of unsuspected genes with ischemic stroke subtypes. Cardioembolic stroke is an ischemic stroke category that includes patients with arterial occlusion processes presumably due to an embolus arising in the heart mainly due to the presence of atrial fibrillation. Atherothrombotic stroke or large vessel atherosclerosis is another ischemic stroke subtype that comprises strokes with >50% stenosis or occlusion of a major brain artery or branch cortical artery, presumably due to atherosclerosis. A meta-analysis of GWAS on ischemic stroke identified the* ZFHX3* gene on chromosome 16q22 as a locus specifically associated with atrial fibrillation and cardioembolic stroke [6, 7]. Additionally, another ischemic stroke-related GWAS showed intriguing results, including the identification of risk variants for atrial fibrillation and cardioembolic stroke on chromosome 4q25 near the* PITX2* gene [8] and for atherothrombotic stroke in the 9p21 locus [9]. Furthermore, an association between large vessel stroke and the* HDAC9* gene on chromosome 7p21.1 was recently identified [10]. Notably, the association of ischemic stroke with* ZFHX3*,* PITX2*, the 9p21 locus, and* HDAC9* was verified in the collaborative METASTROKE study in which these loci were found to be specific to particular stroke subtypes [11].

Inflammatory proteins might therefore play an important role in stroke occurrence. The changes to the atheromatous plaques caused by inflammation mediators in atherothrombotic stroke are associated with vessel occlusion processes and the subsequent development of this subtype of ischemic stroke [12]. Intuitively, it seems that inflammation mediators might be more involved in atherothrombotic stroke compared to cardioembolic or lacunar stroke. However, transcriptomic studies have revealed that inflammation could also have an important role in cardioembolic stroke [13].

It has, therefore, become clear that genetic factors contribute to the development of ischemic stroke and that different genes are involved in the different subtypes of ischemic stroke and they only contribute partially to overall heritability. We hypothesize that most of the genetic studies failed to demonstrate true associations as they did not test ischemic stroke subtypes separately and as they did not take into account the fact that the risk polymorphisms only explain a small part of the ischemic stroke risk.

Our aim is to separately evaluate the genes associated with inflammation processes in the three main ischemic stroke subtypes and to combine the risk polymorphisms into a genetic scoring system in order to evaluate the value of inflammation mediator gene polymorphisms in diagnostic prediction.

## 2. Methods

### 2.1. Study Population

Our discovery cohort and our replication cohort consisted of consecutive Caucasian patients with an ischemic stroke that were admitted to the emergency room of a university hospital. Healthy controls for both cohorts were recruited at the same hospital and were free of ischemic stroke episodes, myocardial infarction, or other cardiovascular diseases.

The discovery cohort (*n* = 1,612; 1,456 ischemic stroke cases and 156 controls) was recruited between July 2005 and May 2009. The replication cohort (*n* = 1,073; 531 ischemic stroke cases and 542 controls) was recruited between November 2000 and May 2005 in a university hospital and the full clinical protocol has previously been published [14].

Etiological stroke subgroups were determined according to the Trial of Org 10172 in Acute Stroke Treatment (TOAST) criteria [15]. In the discovery cohort, 374 ischemic strokes were cardioembolic, 301 were atherothrombotic (large vessel stroke), 226 were lacunar, and 555 were undetermined ischemic stroke subtypes. In the replication cohort, 242 ischemic strokes were cardioembolic, 113 were atherothrombotic, and 176 were lacunar or undetermined ischemic stroke subtypes. Patient information regarding established risk factors, including male gender, smoking, hypertension, diabetes mellitus, and dyslipidemia, was collected. Also, written informed consent was obtained from all subjects, who were all of White European ancestry. In addition, the local ethics committees approved the study.

### 2.2. Genetic Analysis

We identified 68 single nucleotide polymorphisms (SNPs) in 30 genes selected from the literature related to inflammatory pathways associated with stroke (Table 1).

The most relevant candidate genes were selected by manual searching in Pubmed using the keywords “stroke AND inflammation” or “inflammation” for phenotypes and the keywords “polymorphism, SNP, mutation, variant” for polymorphisms. Only articles in English or Spanish were read. Among candidate genes, SNP selection was performed depending on previous literature (the most studied SNPs) and their functional effect, including those with an already known modification in transcription, translation, or protein activity or a hypothetical modification based on an amino acid substitution. Whenever an interesting polymorphism involved more than a single nucleotide change a SNP in perfect linkage disequilibrium was chosen for genotyping.

In addition, we analyzed the whole gene region of the* IL6*,* MMP9*, and* NOS3* genes with tag SNPs using data from the Hapmap project (http://hapmap.ncbi.nlm.nih.gov/). We selected the SNPs using the Tagger computer program in pairwise mode, MAF > 0.1 (minor allele frequency), and linkage disequilibrium *r* ^2^ > 0.8 and with central European population settings.

Genotyping was carried out at CEGEN (Barcelona, Spain), using SNPlex technology (Applied Biosystems, Foster City, California) and GeneMapper 3.5 as the allele-calling algorithm. For quality control, two HapMap samples (NA10860 and NA10861) were included, and their genotype concordance was verified using SNPator (http://www.snpator.org/). All the 68 SNPs achieved the minimum call rate.

### 2.3. Statistical Analysis and Score Generation

Sample size calculation was performed using Ene 2.0 software (Servei d'Estadística Aplicada, UAB, Barcelona, http://sct.uab.cat/estadistica/content/programari-d%27interes). A total of 110 subjects were needed in order to detect SNP frequencies <0.30 in the experimental group (ischemic stroke patients) and <0.15 in the reference group (healthy controls) with a statistical power of 80% and *p* value = 0.05.

Statistical analysis was performed using SPSS software, v.15 (IBM, Chicago, Illinois). Statistical significance for each SNP in the discovery cohort was assessed by chi-square analysis or Fisher's exact test.

Continuous variables were compared by analysis of variance (ANOVA) or Mann-Whitney or Kruskal-Wallis tests.

We generated a predictive score based on logistic regression (LR) and odds ratio (OR) coefficients, using forward stepwise procedure, with a *p* value of 0.05 as the threshold for entry as previously described [14]. Afterwards, to establish clinically relevant cut-off values, we automatically categorized this score into risk groups with the mathematic algorithm chi-squared Automatic Interaction Detector (CHAID), included in SPSS.

## 3. Results and Discussion

### 3.1. Results

The study included a total of 2,685 subjects. The discovery cohort included 1,456 ischemic stroke cases and 156 controls and the replication cohort included 531 ischemic stroke cases and 542 controls. In terms of the TOAST etiology, in the discovery cohort, 374 ischemic strokes were cardioembolic, 301 were atherothrombotic (large vessel stroke), 226 were lacunar, and 555 were undetermined ischemic stroke subtypes. In the replication cohort, 242 ischemic strokes were cardioembolic, 113 were atherothrombotic, and 176 were lacunar or undetermined ischemic stroke subtypes.

No SNPs were found to be associated with atherothrombotic stroke or lacunar stroke after statistical analyses on the discovery cohort. Three SNPs were associated with cardioembolic stroke following a dominant/recessive genetic model (Table 3).

The SNP rs1205 was located in the untranslated region (UTR) of the C-reactive protein (*CRP*) gene region and SNPs rs1800779 and rs2257073 in the nitric oxide synthase-3 (*NOS3*) gene region. When we selected the cardioembolic stroke patients with atrial fibrillation before stroke onset (46.4%), the three SNPs remained associated with cardioembolic stroke with the exception of rs1205 (CC-cases: 40.1%, CC-controls: 51.3%; *p* value = 0.052), rs1800779 (GG-cases: 22.7%, GG-controls: 11.6%; *p* value = 0.033), and rs2257073 (TT-cases: 4.3%, TT-controls: 10.5%; *p* value = 0.042).

Using the OR of the risk factors' genotypes (Table 3) we generated a genetic risk score for cardioembolic stroke:

(1) Genetic  risk  score:1.5×rs1205CT/TT+2.2×rs1800779GG+2.1×rs2257073CT/CC.

This score was associated with the risk of cardioembolic stroke in the discovery cohort (cases: 3.4 points; controls: 2.9 points; *p* value = 0.001). The genetic score was validated in the replication cohort with the cardioembolic stroke group (cases: 3.5 points; controls: 3.08 points; *p* value = 0.017). However, this score was not associated with the risk of atherothrombotic stroke (*p* value = 0.24) or lacunar stroke (*p* value = 0.7). Interestingly, we observed similar results in the undetermined stroke group compared to the cardioembolic stroke cases (cases: 3.3 points; controls: 3.08 points; *p* value = 0.07), although the results were not significant.

Hypertension and sex were the only clinical risk factors associated with cardioembolic stroke (Table 2). When we included these variables in the score, the association with cardioembolic stroke was even more significant (cases: 7.6 points; controls: 6.6 points; *p* value = 8.3 × 10^−05^) and this was replicated in the replication cohort (cases: 5.1 points; controls: 4.2 points; *p* value = 0.002). However, for these two clinical variables the score was also significantly associated with atherothrombotic stroke (cases: 6.1 points; controls: 4.2 points; *p* value = 0.003) and undetermined stroke (cases: 5.1 points; controls: 4.2 points; *p* value = 0.025), although it was not associated with lacunar stroke (*p* value = 0.31).

**Figure 1:**
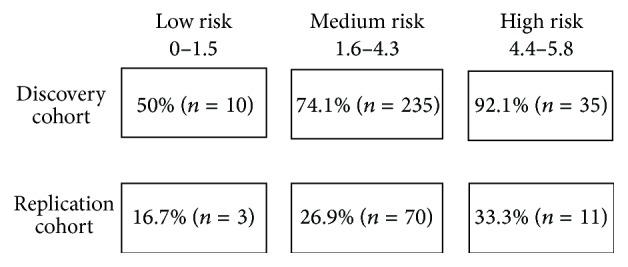
Cardioembolic risk groups based on genetic risk score results. The percentage indicates the percentage of subjects inside the group that presented a cardioembolic stroke.

Using the CHAID method implemented by SPSS software we obtained three different risk groups for cardioembolic stroke and controls depending on the genetic risk score that we generated: a low risk group (score from 0 to 1.5 points), a medium risk group (from 1.6 to 4.3 points), and a high risk group (from 4.4 to 5.8 points). When we classified patients and controls using this classification, 92% of the high risk group presented a cardioembolic stroke (low risk: 50% of subjects with cardioembolic stroke, medium risk: 74.1% of subjects with cardioembolic stroke, and high risk: 92% of subjects with cardioembolic stroke; *p* value = 0.002) (Figure 1).

### 3.2. Discussion

We aimed to combine risk polymorphisms into a genetic scoring system in order to evaluate the diagnostic prediction of polymorphisms of inflammatory mediator genes for ischemic stroke subtypes.

In addition, we aimed to study the role of inflammatory genes in ischemic stroke subtypes in order to establish which ischemic stroke subtype is most greatly influenced by the genetic background of the inflammatory mediator genes.

We generated a genetic risk scoring system for three polymorphisms associated with cardioembolic stroke. The genetic risk score was weighted by the OR of every genotype and was validated in an independent replication cohort. The genetic risk score was not associated with atherothrombotic stroke or with lacunar stroke, the other subtypes of ischemic stroke. Interestingly, we found a trend of association with undetermined stroke. We hypothesize that the trend of association between the score and undetermined stroke is due to a high percentage of undetermined strokes that are in fact cardioembolic strokes that have not been correctly diagnosed. When we generated a new score combining clinical risk factors (sex and hypertension) with genetic risk factors, we observed that this new clinical-genetic score was associated with the cardioembolic stroke subtype in both the discovery and replication cohorts. However, this score was also associated with atherothrombotic stroke. Ischemic stroke subtypes have different genetic risk factors, and we observed that the inclusion of sex and hypertension introduces a confusing factor that reduces the accuracy of the genetic score. The use of these genetic scores could be very useful in the clinical practice to categorize the patients with atrial fibrillation. The genetic scores could detect those patients with the highest risk of suffering a future cardioembolic stroke and consequently initiate a treatment with anticoagulants.

The polymorphisms and inflammatory mediator genes analyzed in this study were only associated with the cardioembolic stroke subtype. This is very interesting as inflammation has been classically more closely linked to atherosclerotic processes than to cardioembolism. During the last few years evidence has been found supporting the hypothesis that inflammation plays a key role in cardioembolic stroke and atrial fibrillation, the main risk factor for cardioembolic stroke [16]. Interestingly when we selected the cardioembolic stroke patients with atrial fibrillation before the stroke, the three SNPs of CRP and NOS3 were still associated with cardioembolic stroke, with very similar results compared to the whole group of cardioembolic stroke patients, indicating an association of these genetic markers with cardioembolic stroke patients with atrial fibrillation and without atrial fibrillation. Moreover, previous transcriptomic studies using blood samples found inflammatory mediator genes played an important role in cardioembolic stroke [13]. Our study found that, in terms of genetics, inflammation also plays a key role in cardioembolic stroke.

The* NOS3* gene codes for nitric oxide synthase (NOS), an enzyme that synthesizes nitric oxide (NO) from L-arginine. NO is a reactive free radical that acts as a biological mediator in several processes, including the migration or proliferation of endothelial cells, platelet aggregation, and leukocyte adhesion. NOS can regulate blood pressure by the synthesis of NO, and it can also regulate metalloproteinase-2 (MMP2) and metalloproteinase-9 (MMP9), which have been associated with angiogenesis and inflammatory processes. In fact, inhibition of NOS increases MMP2 activity [17–19]. Our study found that the rs1800779 GG genotype and the C carriers of the rs2257073 SNP were associated with a higher risk of cardioembolic stroke. The rs2257073 SNP was a tag SNP; however, the rs1800779 SNP of* NOS3* has been associated with an inhibition of the enzyme's activity, causing a decrease in NO production, and it has also been associated with the presence of leukoaraiosis by a previous paper [20]. The GG genotype was associated with a higher risk of leukoaraiosis (a rarefaction of the brain white matter). Interestingly, leukoaraiosis has been associated with inflammation, hypertension, and blood brain-barrier disruption.

C-reactive protein (CRP) is a marker of systemic inflammation that is significantly associated with an increased risk of cardiovascular disease in the general population. CRP has previously been associated with cardioembolic ischemic stroke. Terruzzi et al. [21] studied 648 stroke patients with a first documented cerebral infarction and they measured CRP within the first 6 hours after onset and the CRP levels were then stratified in quartiles. The results showed that CRP quartiles were mostly increased in cardioembolic strokes; this suggests that, in the acute phase of the cerebral infarction, CRP might be a marker of cardioembolism [21]. Another prospective study evaluated a cohort of 2,084 Japanese ischemic stroke patients admitted in the first 7 days of onset; the authors of the study showed that CRP is an independent risk factor in the recurrence of cardioembolic ischemic stroke during the first year after symptom onset [22].

## 4. Conclusions

In summary, we observed that polymorphisms of inflammatory mediator genes were more closely associated with cardioembolic stroke than with other subtypes of ischemic stroke including atherothrombotic stroke. In addition, we generated a genetic risk scoring system to predict the risk of cardioembolic stroke, which we also validated in an independent population. The implementation of genetic scoring systems can be useful in clinical practice to facilitate the prediction of the risk of stroke in healthy people; however, further studies are needed to confirm these results.

## Figures and Tables

**Table 1:** List of polymorphisms and SNPs analyzed.

Gene	SNP	Chromosome
*CD40 *	rs1883832	20
*CRP *	rs1130864	1
*CRP *	rs1205	1
*CRP *	rs1800947	1
*IFNG *	rs2430561	12
*IL10 *	rs1800872	1
*IL10 *	rs1800896	1
*IL13 *	rs1295686	5
*IL1A *	rs1800587	2
*IL1B *	rs1143627	2
*IL1B *	rs1143634	2
*IL1B *	rs16944	2
*IL4R *	rs1801275	16
*IL4R *	rs1805015	16
*IL5 *	rs2069812	5
*IL5RA *	rs2290608	3
*IL6 *	rs1800795	7
*IL6 *	rs1800796	7
*IL6 *	rs1800797	7
*IL9 *	rs2069885	5
*ITGA2 *	rs1126643	5
*MCP1 *	rs1024611	17
*MMP1 *	rs1799750	11
*MMP10 *	rs486055	11
*MMP12 *	rs2276109	11
*MMP13 *	rs2252070	11
*MMP2 *	rs243864	16
*MMP3 *	rs3025058	11
*MMP7 *	rs11568818	11
*MMP8 *	rs1320632	11
*MMP9 *	rs2236416	20
*MMP9 *	rs2250889	20
*MMP9 *	rs2274755	20
*MMP9 *	rs2274756	20
*MMP9 *	rs3787268	20
*MMP9 *	rs3918241	20
*MMP9 *	rs3918248	20
*MMP9 *	rs3918253	20
*MMP9 *	rs3918256	20
*MMP9 *	rs3918278	20
*MMP9 *	rs8113877	20
*NOS2A *	rs1137933	17
*NOS3 *	rs10266564	7
*NOS3 *	rs10275136	7
*NOS3 *	rs12703116	7
*NOS3 *	rs1800779	7
*NOS3 *	rs2070744	7
*NOS3 *	rs2243428	7
*NOS3 *	rs2257073	7
*NOS3 *	rs2257090	7
*NOS3 *	rs2288649	7
*NOS3 *	rs2435608	7
*NOS3 *	rs2435609	7
*NOS3 *	rs2487151	7
*NOS3 *	rs310584	7
*NOS3 *	rs310585	7
*NOS3 *	rs310586	7
*NOS3 *	rs310588	7
*NOS3 *	rs310589	7
*NOS3 *	rs310590	7
*NOS3 *	rs4722204	7
*NOS3 *	rs6952465	7
*SELE *	rs5355	1
*SELE *	rs5361	1
*SELP *	rs6133	1
*TIMP1 *	rs2070584	23
*TNF *	rs1800629	6
*TNFRSF1B *	rs1061622	1

SNP: single nucleotide polymorphism.

**Table 2:** Clinical risk factors analyzed in the discovery and replication cohorts.

	Discovery cases	Discovery controls	*p* value	Replication cases	Replication controls	*p* value
	(*n* = 374)	(*n* = 156)		(*n* = 242)	(*n* = 547)	
Male	50.4% (188)	28.5% (37)	1.4 × 10^−05^	47.5% (115)	42.5% (230)	0.19
Female	49.6% (185)	71.5% (93)	1.4 × 10^−05^	52.5% (127)	57.5% (311)	0.19
Presence of hypertension	63.1% (231)	40% (52)	4.7 × 10^−06^	59.8% (143)	44.2% (239)	5.5 × 10^−05^
Current smoker	13.8% (49)	14.6% (19)	0.77	18.3% (43)	12.6% (68)	0.036
Presence of diabetes mellitus	22% (80)	14.6% (19)	0.07	19.9% (48)	8.2% (44)	2.7 × 10^−06^
Presence of dyslipidemia	34.6% (125)	27.7% (36)	0.15	25.8% (62)	28.7% (155)	0.41

The significant results are in bold.

**Table 3:** Results of the significant genotypes associated with cardioembolic stroke in the discovery cohort.

Gene	Chr	SNP	Position	Genotype	Cases discovery (*n* = 374)	Controls discovery (*n* = 156)	OR (CI 95%)	*p* value discovery	Cases replication (*n* = 242)	Controls replication (*n* = 547)	OR (CI 95%)	*p* value replication
*NOS3 *	7	rs1800779	150992855	GG versus (AG/GG)	22.4%	11.6%	2.2 (1.11–4.38)	0.021	21.6%	19.4%	1.14 (0.64–2.03)	0.64

*NOS3 *	7	rs2257073	151186200	TT versus (CT/CC)	5.2%	10.5%	0.46 (0.22–0.95)	0.033	5.4%	7.8%	0.67 (0.24–1.11)	0.43

*CRP *	1	rs1205	159712443	CC versus (CT/TT)	40.9%	51.3%	0.65 (0.44–0.96	0.032	40.4%	45.7%	0.8 (0.58–0.95)	0.18

Chr: chromosome, OR: odds ratio, and CI: confidence interval.
